# The Utility of an Epidural Steroid Injection for the Treatment of Idiopathic Brachial Neuritis

**DOI:** 10.7759/cureus.57211

**Published:** 2024-03-29

**Authors:** Zachary Dickey, Navneet Sharma

**Affiliations:** 1 Physical Medicine and Rehabilitation, Edward Via College of Osteopathic Medicine, Monroe, USA; 2 Physical Medicine and Rehabilitation, Green Clinic, Ruston, USA; 3 Rehabilitation, Ruston Regional Specialty Hospital, Ruston, USA

**Keywords:** brachial neuritis, sports medicine, interventional pain medicine, physical medicine and rehabilitation (pm&r), brachial plexus anatomy, acute pain, shoulder joint pain, parsonage turner syndrome, epidural injections

## Abstract

Idiopathic brachial neuritis is an uncommon disorder that predominately affects the superior and middle trunks of the brachial plexus. Severe throbbing and aching shoulder pain is initially present for a period of days to weeks, followed by severe weakness and atrophy that can develop for an extended period of months to years. There are currently no known treatments for brachial neuritis, with the standard of care consisting of analgesics and corticosteroids, which typically provide minimal to no benefit in most cases. In this case, we will present a case of a patient who was diagnosed with idiopathic brachial neuritis and underwent an interlaminar epidural steroid injection (ESI) for treatment. Following treatment with the ESI, the patient had a subsequent resolution of symptoms. This case underscores the value of early recognition for the diagnosis of brachial neuritis and the utility of an ESI as a treatment option, thus preventing long-term pathological sequalae. To our knowledge, this is the first known reported case to have successfully cured brachial neuritis.

## Introduction

Idiopathic brachial neuritis, also known as Parsonage-Turner syndrome, is an uncommon but often misdiagnosed disease with asymmetric involvement of the brachial plexus [1]. Clinical presentation of brachial neuritis is acute in nature and presents with varying degrees of severe throbbing and radiating shoulder pain unassociated with trauma, followed by a period of paresis and atrophy of the upper extremity and shoulder girdle [2-4]. The most frequently affected muscles are those innervated by nerves branching from the superior and middle trunks of the brachial plexus [2-5]. Given the varying clinical presentation [5] and lack of diagnostic criteria, the diagnosis is one of exclusion; however, proper history and physical exam allow one to include brachial neuritis within a differential diagnosis. There are currently no known curative treatments for brachial neuritis and its prognosis is extremely variable [6]. Here, we will present a case of an early diagnosis of idiopathic brachial neuritis treated through an interlaminar epidural steroid injection (ESI).

## Case presentation

**Figure 1:**
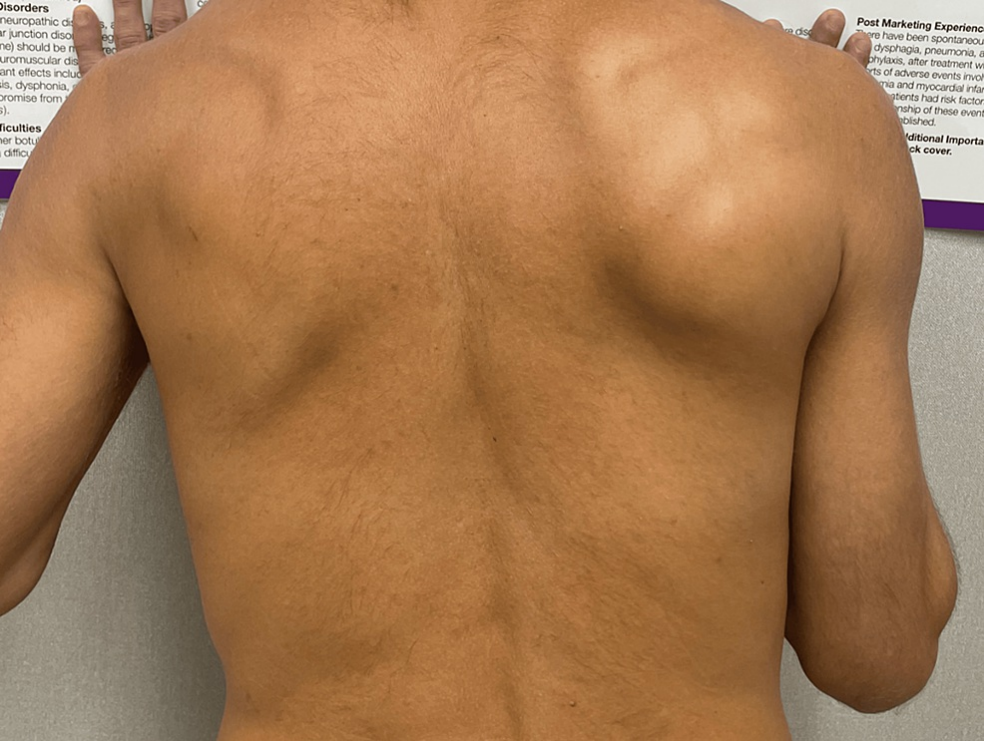
Winging of the right scapula observed upon physical exam and wall push.

A 40-year-old male presented to the clinic with severe acute shoulder pain. The patient is a healthy and active runner who described his shoulder pain as beginning spontaneously before bed the previous evening. The patient described no associated trauma to the affected shoulder, no change in exercise regimen, nor any recent illness or vaccination. Severe guarding of the joint was initially observed with limited to no movement of the shoulder joint. Initial inspection of the joint revealed a depressed right shoulder followed by lateral winging of the right scapula upon flexion of the shoulder (Figure 1). Palpation revealed extreme tenderness to the infraspinatus, teres minor, teres major, and thoracic paraspinals. Exacerbation of pain limited any active range of motion (ROM) with limited passive abduction, adduction, and external and internal rotations. The sensation was intact bilaterally with normal active reflexes. Muscle strength testing was limited due to pain.

Following the initial history and physical exam, trigger point injections were completed using 8 ml of bupivacaine HCl 0.25% and 1 ml of methylprednisolone (DEPO-Medrol) 40 mg/ml within the thoracic paraspinal musculature, infraspinatus muscle, and subscapular bursa. The patient was prescribed methylprednisolone tablets (Medrol Dosepak) and was sent for X-ray imaging, which was ultimately unremarkable.

**Figure 2:**
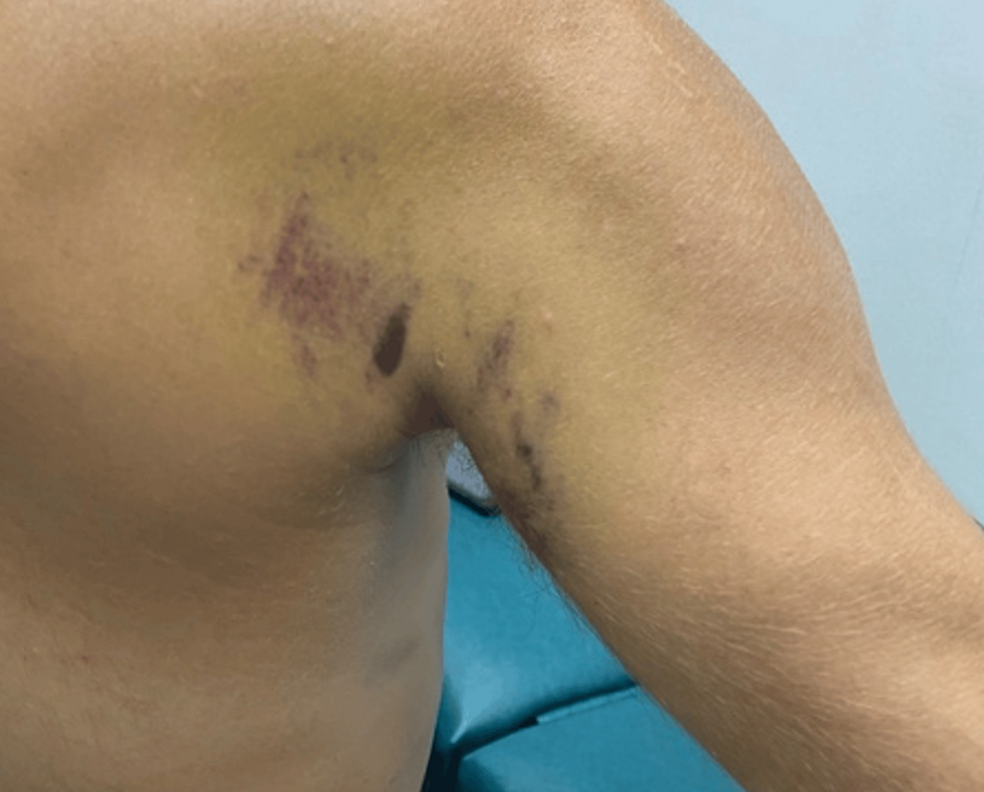
Ecchymosis observed in the right shoulder six days following onset of symptoms.

Six days following the initial encounter, the patient was once again evaluated. The pain had decreased from an initial sharp and stabbing 10/10 to a more bearable 4/10; however, guarding of the shoulder joint was still noted, and exacerbation of pain still limited any active movement. Ecchymosis and bruising were now observed along the posterior shoulder (Figure 2). No atrophy was observed; however, the right posterior shoulder girdle did show decreased bulk and tone.

Tenderness was palpated along the infraspinatus, teres minor, and teres major, with edema noted to be present. Although passive ROM was now equal bilaterally, the pain continued to limit active ROM and muscle strength. 

Osteopathic manipulative medicine (OMM) was performed and aimed at increasing ROM and freeing the joint and musculature of any fascial restrictions. Myofascial release was performed on the thoracic paraspinals to release fascial adhesions, traction techniques were used to free the glenoid capsule, and manipulation of an exhaled first rib was used to potentially free any entrapment of nerves traveling through the anterior and middle scalene. Manipulation resulted in a greater degree of active flexion and adduction of the shoulder following treatment.

**Figure 3:**
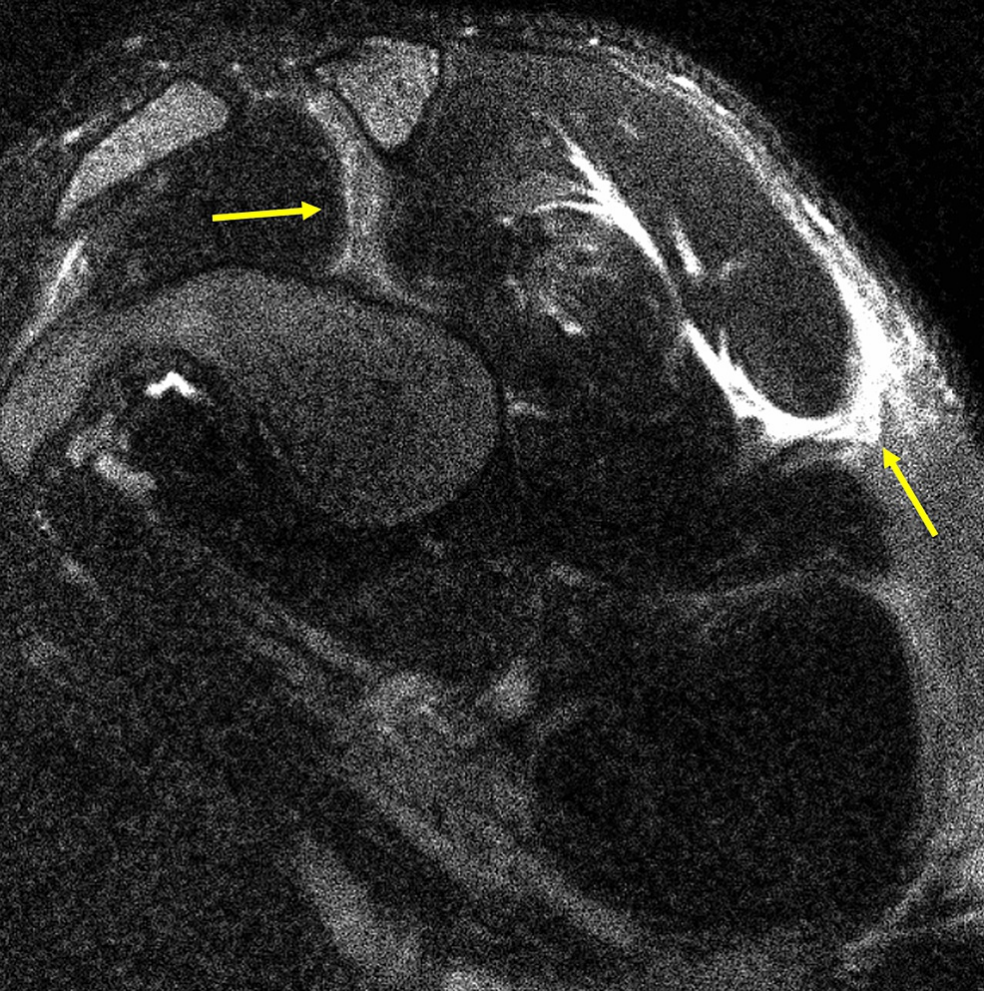
MRI T2-weighted image showing intramuscular edema within the shoulder girdle musculature.

An MRI of the shoulder was ordered and revealed intramuscular edema within the supraspinatus, infraspinatus, teres minor, subscapularis, and teres major (Figure 3). The teres minor and teres major were noted to display muscle fiber disruption. A cervical MRI revealed mild degenerative changes with mild neural foraminal narrowing at C5-C6 on the right and mild left-sided neural foraminal narrowing at C7-T1.

**Figure 4:**
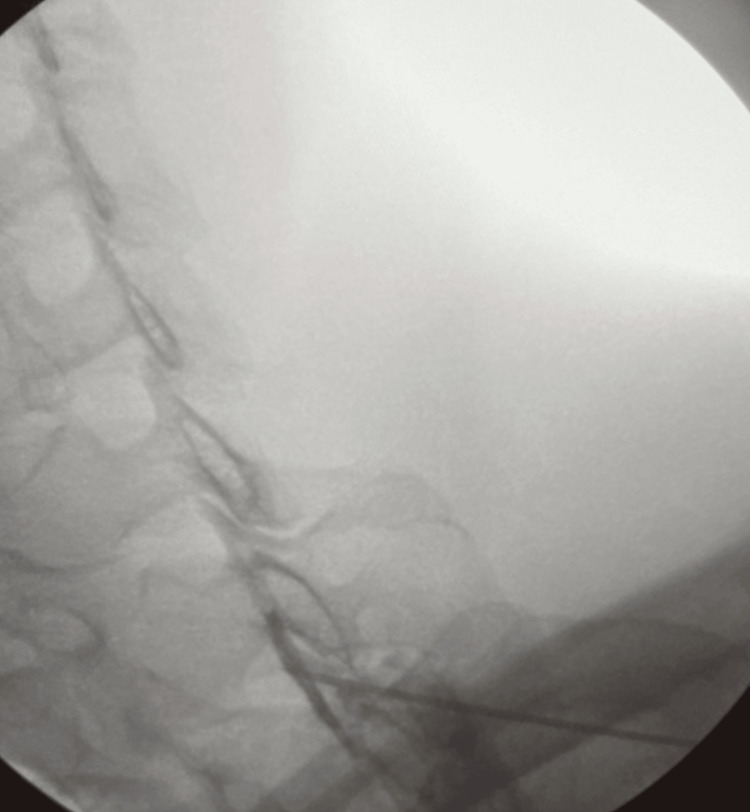
Contrast confirmation of the needle placement within the T1/T2 epidural space.

Nine days following the onset of symptoms and initial encounter, an interlaminar epidural steroid injection with a right paramedian approach was performed at the levels of T1-T2. The patient was locally anesthetized with lidocaine HCl (Xylocaine) 1% 9 ml and sodium bicarbonate 1 ml with 10 mg of dexamethasone injected into the epidural space. Under fluoroscopy guidance, the patient did not show an ideal approach at the desired C7-T1 injection level due to significant narrowing of the interlaminar space. Therefore, the injection was given at the T1-T2 level with the thought of cranial spread allowing the steroid to reach the desired C5, C6, and C7 spinal levels. Through the confirmation of contrast, however, it was observed that the spread may have made it to C7 but not as cranially as C5 (Figure 4).

Following the ESI, the patient reported an immediate improvement in a resting pain scale, from 4/10 to 0/10. The patient regained full active ROM with moderate pain observed at 90 degrees of both flexion and abduction. Prior to injection, the patient had 0 degrees of pain-free active ROM. Strength improved to 5/5 in all ranges of motion of the shoulder joint and the patient was able to resume their exercise regimen and begin strength rehabilitation. The patient reported subsequent improvement in shoulder pain and movement at follow-up visits.

## Discussion

Etiology

Idiopathic brachial neuritis is an uncommon disorder that can sometimes be preceded by a viral infection or vaccination, but often, patient history reveals neither [2]. Viral infection is the most commonly reported etiology associated with preceding symptoms of brachial neuritis; however, it was still only associated with less than a quarter, 50, of the cases reported by Alfen et al. when reviewing 246 cases of brachial neuritis [5]. Vaccination made up another five of those cases [5]. Interestingly, there has recently been a significant number of reported cases of brachial neuritis following both COVID-19 vaccination and COVID-19 infection, further suggesting an autoimmune precedent [7-13]. One systematic review analyzed 68 patients, 32 post-vaccination and 34 post-infection with COVID-19, who experienced brachial neuritis following vaccination or infection [7]. Another recent report noted 42 specific cases reported following vaccination [12]. Without an updated systematic review of reported brachial neuritis cases, one cannot conclude the most common etiology; however, in the review of literature presented here, COVID-19 vaccination and infection are now leading associated causes of reported cases of brachial neuritis.

This associated autoimmune reaction has been proposed to be a form of molecular mimicry or bystander activation of immune-mediated processes [12]. mRNA vaccines, such as the COVID-19 vaccine, illicit a strong type 1 interferon response, activating CD8+ T cells and enhancing the response to the vaccine, but doing so can also lead to autoimmune reactions [14]. Two studies have shown the role of autoimmunity in the pathophysiology of brachial neuritis, although neither has been replicated [15,16]. Sierra et al.showed that lymphocytes from patients with brachial neuritis have increased blastogenic activity in cells isolated from the brachial plexus compared with cells from the sacral plexus [15]. Vriesendorp et al. showed complement-fixing antibodies to myelin within the brachial plexus in the acute phase of the disease process [16]. Given the frequent misdiagnosis and low reported number of cases of brachial neuritis, however, establishing a true correlation is difficult.

Strenuous exercise has also been associated as an antecedent event to developing brachial neuritis [5]. In this case, we report that the patient is an avid runner and did exercise prior to developing symptoms; however, the patient described the exercise as a part of their daily routine. It is unknown whether any sort of exercise alone is enough to contribute to the pathogenesis of brachial neuritis or whether the exercise must be out of proportion to one’s normal routine to be associated with the stress needed to induce brachial neuritis. Other antecedent events include surgery, radiation, pregnancy and parturition, stress, treatment with interferon, and trauma [3,5].

Clinical presentation

**Figure 5:**
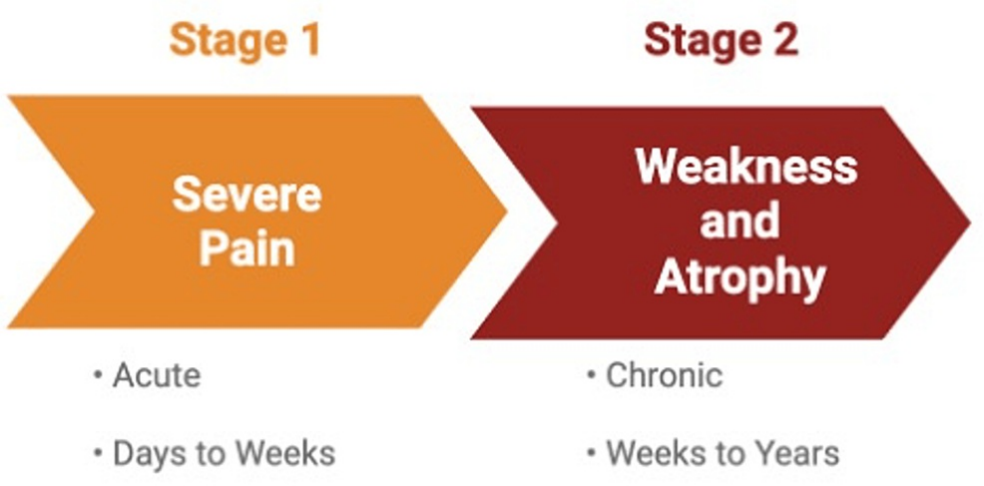
Brachial neuritis clinical timeline. This figure was created via BioRender by Zachary Dickey.

While the etiology remains unclear, the presentation does appear to follow a consistent timeline that can be categorized into two phases (Figure 5). The first stage is characterized by an acute presentation of severe throbbing and aching shoulder pain that can last anywhere from days to weeks [2-4]. The second stage is characterized by significant weakness and atrophy that can last weeks to years [2-4]. Alfren et al.’s review of 246 patients with brachial neuritis showed that the average initial pain phase lasted approximately four weeks, with two-thirds of patients still experiencing weakness and paresis at a three-year follow-up [5]. In the majority of cases, severe pain is the first symptom and typically presents in the evening, often waking patients from sleep [5].

**Figure 6:**
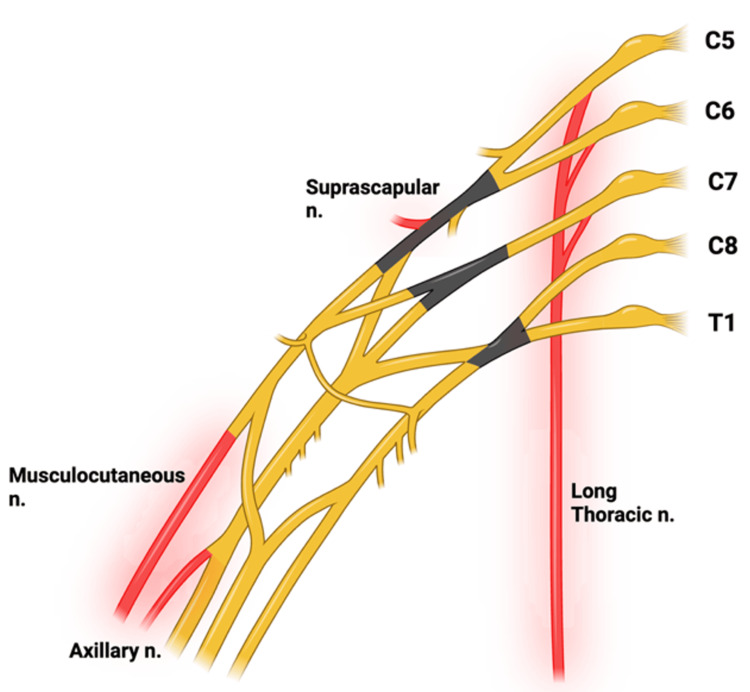
The most affected nerves from brachial neuritis are highlighted in red. The trunks of the brachial plexus are highlighted in black, with the most affected nerves described above branching from the superior and middle trunks. This figure was created via BioRender by Zachary Dickey.

The most common muscles affected are those innervated by nerves originating from the superior and middle trunks of the brachial plexus [2-5]. Of these, the most affected nerves include, but are not limited to, the suprascapular, axillary, long thoracic, and musculocutaneous nerves [2,4,5] (Figure 6). Therefore, the infraspinatus, supraspinatus, serratus anterior, teres minor, deltoid, and biceps will be the most affected muscles and thus should stand out on physical exam. Due to the effect of the long thoracic nerve, winging of the scapula may be observed on physical exam.

Differential diagnosis

When evaluating a patient presenting with idiopathic brachial neuritis, a differential diagnosis can include cervical radiculopathy, rotator cuff strain, mononeuropathy, thoracic outlet syndrome, and an obstructing tumor. While the diagnosis is one of exclusion and typically a challenging one to make, a proper physical exam allows one to rule out less likely diagnoses. To aid in proper diagnosis, it is important to recognize the difference in the clinical presentation of these pathologies. Cervical radiculopathy will typically present with radiation of pain in the distribution of a single nerve root, while brachial neuritis will involve neurological deficits in multiple nerve root distributions [3]. In cervical radiculopathy cases, EMG/NCS along with MRI will also show diagnostic abnormalities, whereas acute neuropathic etiologies, such as brachial neuritis, will not show diagnostic abnormalities within the acute phase [2]. Rotator cuff strains will often include acute onset, localized pain, and worse with movement and be associated with a traumatic event [2,3]. When presented with acute shoulder pain or neuropathic symptoms compromising multiple dermatome and myotome patterns in the absence of trauma, brachial neuritis should be considered.

Diagnostic evaluation

Initial chest radiographs may be of benefit to rule out any underlying pathology, such as a tumor affecting the long thoracic nerve, while MRI may be of use in ruling out cervical pathology, rotator cuff tendinopathies, or other potential pathologies.

Outside of ruling out potential underlying pathology, MRI appears to be of clinical significance in the diagnosis of brachial neuritis. An early detectable sign of denervation of musculature is high signal intensity on T2-weighted images due to diffuse edema within the affected musculature [17]. In the subacute and chronic phases, T2 images will still be remarkable for diffuse edema; however, intramuscular T1-weighted images will now be remarkable due to fatty infiltration from the development of atrophy [17]. MRI of the brachial plexus may show a thickened and hyperintense brachial plexus trunk [5,12] but is not definitive. A review of 50 patients who underwent MRIs of the brachial plexus showed only three of those studies to be remarkable for a hyperintense brachial plexus [5]. If available, it may be recommended that those looking to explore imaging of the brachial plexus undergo magnetic resonance neurography (MRN), which is more sensitive to detecting brachial plexus etiologies [18]. Hence, while these studies may have diagnostic potential, one must also weigh the cost-to-benefit ratio for the patient in the number of imaging studies ordered.

Electrodiagnostic studies in patients with brachial neuritis will show characteristic findings of axonal damage in the sub-acute and chronic phases [5]. In the acute phase, as previously mentioned, electrodiagnostic tests, such as nerve conduction studies (NCS) or electromyography (EMG), will not be helpful due to the fact that Wallerian degeneration takes up to four weeks to show pathologic axonal damage; therefore, acute neuropathic pathologies will fail to result in positive findings. Patients with brachial neuritis who undergo EMGs after the initial four-week period typically will reveal positive sharp waves and fibrillations acutely, and if done in the chronic phase, EMG may show chronic denervation with signs of early reinnervation and polyphasic motor unit potentials [5].

Current treatment modalities

Current general recommendations for the treatment of brachial neuritis include a symptomatic approach that includes analgesics, corticosteroids, and muscle relaxers; however, there are no specific treatment options for brachial neuritis [6]. A Cochrane review completed by Alfen et al. showed that there is no significant evidence to support any form of treatment for brachial neuritis, with no cases described to successfully treat and or cure brachial neuritis [6].

Case discussion

A detailed and thorough history and physical examination (H&P) is essential for ruling out potential pathology and successfully making the diagnosis of idiopathic brachial neuritis. By not properly diagnosing brachial neuritis, a doctor may put a patient through unnecessary and invasive procedures [3]. Early diagnosis is helpful in guiding a multidisciplinary treatment approach and in reassuring the patient that they have a relatively good prognosis, although recovery may take up to several years [5,19]. In a review of 99 patients, recovery at one year was just 36%, 75% at two years, and 89% at three years [19]. Thus, while long-term recovery is favorable, the recovery process is not predictable and can be detrimental to both the physical and mental health of a patient.

Following prompt recognition and diagnosis of brachial neuritis, investigations into successful treatment modalities become imperative in preventing long-term pathological sequelae. Therefore, we sought to explore the utility of an ESI for the treatment of brachial neuritis.

Due to the fact that oral corticosteroids have minimal to no significant effects on the prognosis of brachial neuritis [6], we sought to better understand and treat the root cause of the disease. Understanding that this idiopathic disease process stems predominately from the superior and middle trunks of the brachial plexus, the safest way to get medications directly to the desired area of interest is by entering the epidural space and allowing the medication to spread cephalo-caudal to affect the desired nerve roots. The commonly accepted practice is the spread of corticosteroid 1-2 levels both cephalad and caudad to the targeted level of injection. Therefore, a C7-T1 interlaminar approach would ideally spread cephalad to the affected C5 and C6 nerve roots and to the C7 nerve root.

Interestingly, in the patient treated in our case, the C7-T1 interlaminar approach was not ideal due to the narrowing of the interlaminar space, so the T1-T2 interlaminar space was targeted. The patient did report immediate relief following the ESI with subsequent improvement in the following days. Had the patient not had as significant of a benefit as predicted, repeat injection would have been completed with the patient in a more flexed position to better access the C7-T1 interlaminar space; alternatively, a transforaminal approach at C5 and C6 would have been used. Given the significant improvement seen in this patient, it is best predicted that the steroid did reach the affected spinal nerves.

Following the injection, the patient immediately resumed physical activity and rehabilitation of the affected shoulder. It is possible that resuming physical activity and moving the shoulder prevented further sequelae of the disease and prevented the chronic weakness and atrophic state. Given that this is the first reported case to utilize an ESI for the treatment of brachial neuritis and the injection was given prior to the emergence of any chronic sequelae, further investigation will be needed for a more definitive answer to the relationship between ESI and rehabilitation in the healing of brachial neuritis. Physiotherapy does appear to be of benefit in the rehabilitation of brachial neuritis [20]; however, further studies would be needed to draw a more significant conclusion. Due to its benefits, we would recommend it regardless.

## Conclusions

This case underscores the value of early recognition along with proper history and physical examination for the diagnosis of brachial neuritis and the utility of an ESI as a treatment option, thus preventing long-term pathological sequalae. We present a case that utilized an interlaminar ESI with a right paramedian approach to successfully treat a case of brachial neuritis. While this is only a case report, this report should suggest the investigation into trials for the use of ESIs for the treatment of brachial neuritis. To our knowledge, this is the first known reported case to have successfully cured brachial neuritis.
